# Advancements in research on the anti-aging effects and mechanisms of flavonoids in natural products

**DOI:** 10.3389/fmed.2025.1637992

**Published:** 2025-08-19

**Authors:** Wan-yu Li, Liang Zhang, Xing-wen Xie, Xiao-feng Shi, Qu-huan Ma

**Affiliations:** ^1^College of Pharmacy, Gansu University of Traditional Chinese Medicine, Lanzhou, China; ^2^Pharmaceutical Research Institute of Gansu Academy of Medical Sciences, Lanzhou, China; ^3^Gansu Medical College, Pingliang, China; ^4^Affiliated Hospital of Gansu University of Traditional Chinese Medicine, Lanzhou, China

**Keywords:** flavonoids, anti-aging effects, different models, antioxidants, mechanism of action, research progress

## Abstract

In recent years, with the continuous development of social productivity and improvements in living standards, the issue of population aging has intensified. As the elderly are susceptible to cardiovascular diseases, osteoporosis, Alzheimer’s disease, and other conditions, the economic and resource burdens associated with treating, caring for, and supporting this demographic rise with their growing numbers, imposing significant pressure on societal development. In light of these challenges, research on natural products possessing anti-aging properties for treating aging-related chronic diseases has gained prominence. Flavonoids, a class of natural bioactive compounds abundantly found in plants, exhibit diverse pharmacological effects, including antioxidant, anti-inflammatory, and anti-osteoporosis activities. Therefore, studying the anti-aging effects of flavonoids is of significant value. This paper reviews recent research on the anti-aging effects of flavonoids by searching databases including CNKI, PubMed, Web of Science, and Google Scholar. The findings are summarized based on research subjects (C. elegans, animal models, and cells), effects, and mechanisms, to provide references for future in-depth research on the anti-aging effects of flavonoids.

## 1 Introduction

**FIGURE 1:**
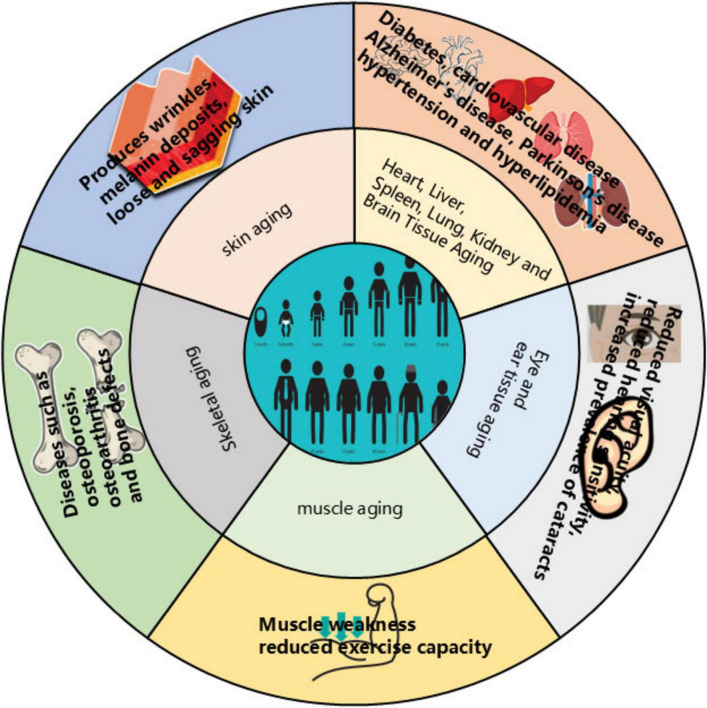
Effects of aging on the organism.

Aging represents an inevitable biological process characterized by a progressive decline in metabolic function and deterioration of organs and tissues. Due to this systemic degradation, the elderly exhibit heightened susceptibility to cardiovascular and neurological disorders, including hypertension, hyperlipidemia, diabetes, and Alzheimer’s disease. Visible manifestations of aging encompass sensory decline (hearing loss, impaired vision), integumentary changes (skin sagging, increased wrinkles), diminished physical fitness and endurance, and gradual muscle weakening. Additionally, elderly women face an increased risk of osteoporosis (1, 2) (see Figure 1). Currently, population aging is intensifying globally, notably in China and numerous other nations (3), Driven

by persistently declining birth rates and steadily increasing life expectancy, the global population aged 65 and above is projected to reach 1.1 billion by 2035, constituting 13% of the world’s population. This demographic shift will significantly reduce the proportion of younger individuals. Developed countries are already confronting substantial labor shortages, while developing nations face unprecedented pressures and challenges from accelerated aging. The impact permeates multiple societal domains, including employment, healthcare, economic systems, and service provision. Consequently, ensuring comfortable aging while alleviating the economic burden on younger generations and mitigating associated socioeconomic and healthcare pressures has emerged as an urgent societal imperative. Given the strong association between aging and chronic diseases such as cardiovascular disorders and Alzheimer’s disease, elucidating the aging process is fundamental to understanding these conditions. Therefore, the development of safe, effective, environmentally sustainable, and economically viable anti-aging therapeutics and medical products is critically important.

**FIGURE 2:**
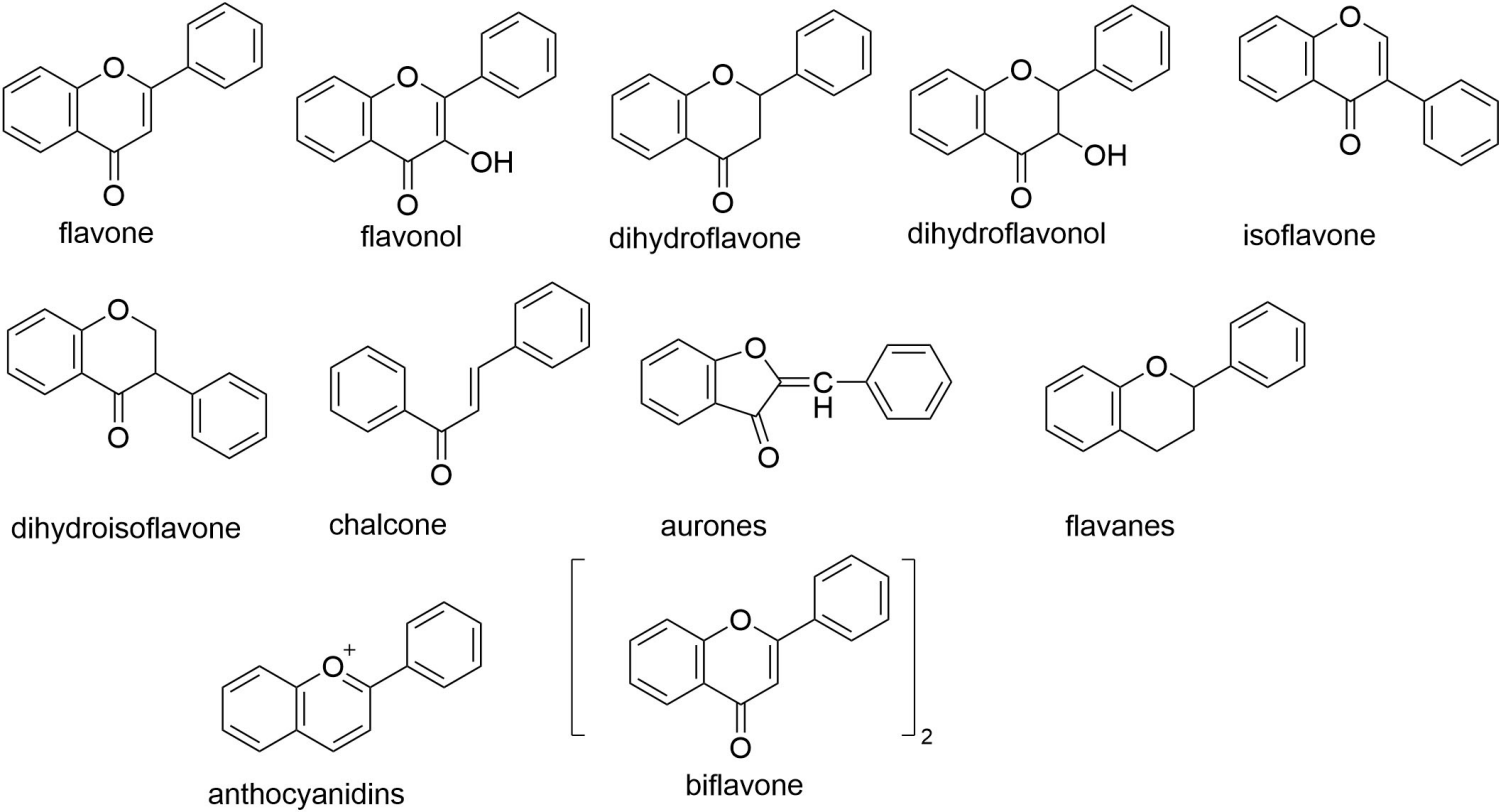
Flavonoid parent nucleus.

Flavonoids comprise a class of compounds characterized by a 2-phenylchromone (flavone) core structure. They are primarily classified into core subclasses: flavanones, flavanols, dihydroflavonoids, dihydroflavonols, isoflavonoids, dihydroisoflavonoids, chalcones, aurones, flavones, and anthocyanins (see Figure 2 for parent nucleus structures). Functionally, flavonoids exist predominantly as glycosides (bound to sugar moieties), with a minority occurring as free aglycones. As ubiquitous plant secondary metabolites, flavonoids are distributed throughout stems, leaves, flowers, fruits, and seeds. Their presence contributes to the vibrant pigmentation of many common foods and botanicals, including citrus fruits, legumes, and medicinal herbs such as liquorice, black goji berries, and ginger (4). This widespread occurrence underscores their status as green, safe, inexpensive, and readily accessible compounds. Modern pharmacological research confirms flavonoids possess significant antioxidant activity—a property critically linked to aging and age-related metabolic diseases (5–7), Accumulating evidence further demonstrates their therapeutic potential against diabetes, neurodegenerative disorders, cardiovascular diseases, and skeletal system pathologies (8–11). Consequently, global research efforts increasingly employ extraction, separation, purification, and synthesis techniques to obtain flavonoid fractions or specific compounds for anti-aging investigations (12), highlighting their substantial promise in this field.

**FIGURE 3:**
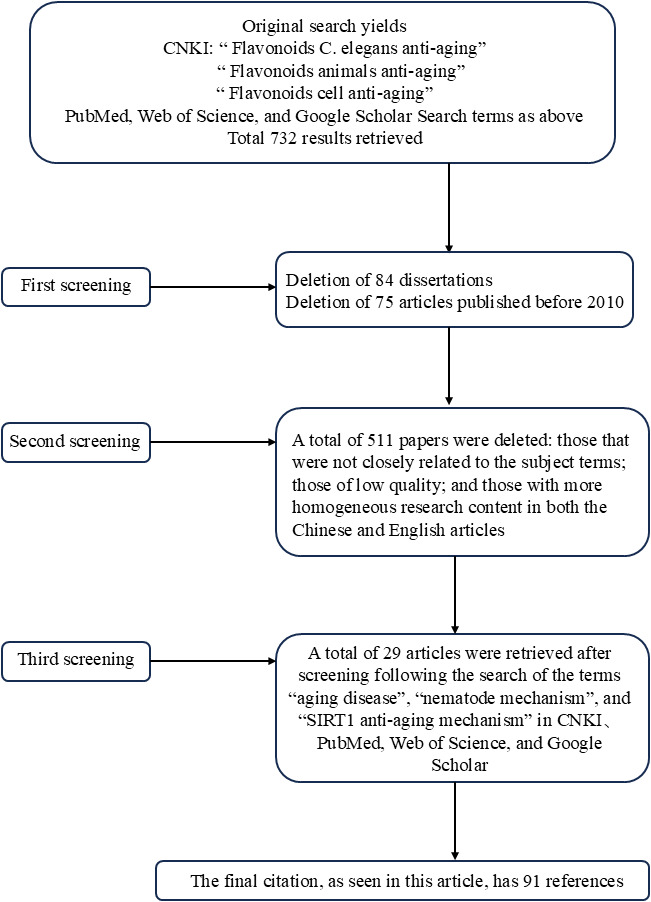
Literature retrieval strategy.

While previous reviews have outlined flavonoids’ anti-aging bioactivities, these often emphasize specific roles and mechanisms (13, 14), with relatively limited systematic organization. Recent advances in model organism applications have accelerated research in this domain. This review synthesizes findings from the most extensively studied anti-aging models—C. elegans, rodent species (rats/mice), and cellular systems—to systematically summarize flavonoid effects and mechanisms. We searched the CNKI, PubMed, Web of Science and Google Scholar databases using relevant keywords, as shown in Figure 3. This structured approach aims to provide future investigators with conceptual frameworks, stimulate novel research directions, and enhance contextual understanding of existing data.

## 2 Studies on the anti-aging effects of flavonoids based on different models

### 2.1 Anti-aging effects of flavonoids in *Caenorhabditis elegans* models

As eukaryotic organisms, C. elegans share skin, muscular, digestive, nervous, and reproductive system homology with higher organisms, along with significant genetic conservation—including with humans. Their short lifespan and high reproductive capacity have established C. elegans as a fundamental model for longevity research since the 1980s (15). making it a cornerstone organism in aging studies. The anti-aging effects and mechanistic pathways of flavonoids in this C. elegans model are summarized in Table 1.

**TABLE 1:** Anti aging effects and mechanisms of natural flavonoids in models of *Caenorhabditis elegans.*

material	Flavonoid classification	Effect	Mechanism	References
Flavonoids of peony petal	Total flavonoids	Lifespan of C. elegans↑	Oxidative stress damage↓	(16)
Flavonoids of onion	Total flavonoids	Lifespan of C. elegans↑	Oxidative stress damage↓, daf-2, old-1, osr-1 and sek-1↑	(17)
Flavonoids of finger citron	Total flavonoids	Good free radical scavenging ability *in vitro*, increasing the average life span of C. elegans by 31.26% and the maximum life span by 26.59%	Oxidative stress damage↓, SOD and CAT↑, ROS and MDA↓	(18)
Flavonoids of jujube seed	Total flavonoids	Good free radical scavenging ability *in vitro*, lifespan of C. elegans↑, resistance to heat stress in C. elegans↑	Oxidative stress damage↓, Aβ↓	(19)
Flavonoids of mung bean coat	Total flavonoids	Lifespan of C. elegans↑, resistance to heat stress in C. elegans↑	Oxidative stress damage↓, ROS↓, mTOR related genes let-363, rsks-1 and daf-15mRNA↓, regulates histone modifications	(20)
Total flavonoids in stems and leaves of *Glycyrrhiza uralensis* Fisch.	Total flavonoids	Lifespan of C. elegans↑, no effect on nematode fertility	Oxidative stress damage↓, ROS↓, antioxidant activity↑, daf-16 and skn-1↑	(21)
Flavonoids of ginkgo seed	Total flavonoids	Lifespan of C. elegans↑, athletic ability↑, no effect on C. elegans fertility, resistance to heat stress in C. elegans↑	Oxidative stress damage↓, daf-16↑, autophagy↑, Aβ↓, ROS↓, lipofuscin↓	(22)
Epicatechin	Flavanes	Lifespan of C. elegans↑, resistance to heat stress in C. elegans↑	Oxidative stress damage↓, antioxidant activity↑, lipofuscin↓, daf-16 RNA, gst-4, hsp-16.2 and hsp-70↑, regulation of insulin/IGF-1 signaling pathway	(23)
4′-O-methylepicatechin	Flavanes	Lifespan of C. elegans↑, no effect on C. elegans fertility, C. elegans swallowing velocity↑	Oxidative stress damage↓, C. elegans metabolism↑	(24)
Flavonoids of *Aronia melanocarpa*	Total flavonoids	Lifespan of C. elegans↑, C. elegans motility↑	Oxidative stress damage↓, ROS↓, antioxidant activity↑, acts by regulating the pmk-1 pathway	(25)
Total flavonoids from *Acer truncatum* samara and leaves	Total flavonoids	Lifespan of C. elegans↑	Oxidative stress damage↓, lipofuscin↓, antioxidant activity↑, inhibition of the insulin/IGF-1 signaling pathway	(26)
Phlorizin	Dihydrochalcone	Lifespan of C. elegans↑, no effect on C. elegans fertility, stress resistance of C. elegans to ultraviolet stimuli↑, C. elegans motility↑	Oxidative stress damage↓, ROS↓, autophagy↑, hsp-16.2, sod-3↑, daf-16 nuclear localization↑	(27)
Phloretin	Dihydrochalcone	Lifespan of C. elegans↑, C. elegans motility↑, resistance to stress↑	Oxidative stress damage↓, antioxidant activity↑, harmful free radical↓, lipofuscin↓, daf-2 and age-1 mRNA↓, daf-16 and sir-2.1 mRNA↑, section IIS signaling pathway and sir2.1 signaling pathway	(28)

Compared to other model organisms, such as higher animals and cells, Caenorhabditis elegans offers advantages including genetic stability, a simple individual structure, a short life cycle, a large population size and low operational costs. It has unique applications in the study of aging and genetic diseases. However, it has limitations in terms of aging phenotypes when compared to higher animals, making it difficult to simulate human skin aging or neurodegenerative disease phenotypes. There are also fundamental differences between C. elegans and higher organisms in terms of physiology and living environments. Therefore, while it is highly promising for the initial screening of flavonoid compounds for anti-aging effects, further validation through animal and cell models is necessary for in-depth research.

### 2.2 Anti-aging effects of flavonoids in animal models

The D-galactose-induced subacute aging model is widely employed in animal studies. High-dose D-galactose administration generates excessive reactive oxygen species (ROS), inducing oxidative stress that exacerbates mitochondrial dysfunction, apoptosis, and tissue damage (29–31). Owing to its procedural simplicity and minimal adverse effects, this model has gained prominence in aging research (32). Flavonoid compounds tested in animal models, along with their specific actions and mechanisms, are detailed in Table 2.

**TABLE 2:** Anti aging effects and mechanisms of natural flavonoids in animal models.

Material	Flavonoid classification	Modeling method	Effect	Mechanism	References
Flavonoids of *Anoectochilus roxburghii*	Total flavonoids	18-month-old naturally aging mice	Learning memory capacity of aging mice↑, apoptosis in mouse hippocampal neuronal cells↓	Oxidative stress damage↓, ROS↓, Number of SA-β-gal positive cells↓, expression of P53 and its downstream signaling molecules↓, SRIT1 and Bcl-2/Bax↑, apoptosis and inflammation↓	(33)
Flavonoids from *Bacopa floribunda*	Total flavonoids	Single bilateral intraventricular injection of Aβ 1-42	Antioxidant ability↑, microglioma and neuroinflammation induced by aβ 1-42↓	Oxidative stress damage↓, SOD↑, MDA↓	(34)
Flavonoids of brown black wolfberry	Total flavonoids	Subcutaneous D-galactose	Amelioration of weight loss in aging mice, immunity↑, lifespan in aging mice↑	Antioxidant activity↑, MDA↓, number of vertebrate cells in brain ↑	(35)
Quercetin	Flavonol	SAM-P8 and SAM-R1 rapid aging mice	Learning memory capacity of aging mice↑, hippocampal oxidative stress damage in aging mice↓, prevention of neuroinflammation in aging mice	SIRT1↑, NLRP3↓, improve neuroinflammation in aging mice by regulating the sirtuin1/NLRP3 pathway	(36)
Proanthocyanidins	Flavanes	Gastric thyroxine	Improvement of cognitive impairment and learning memory in aging mice	Oxidative stress damage↓, antioxidant activity↑, MDA↓, immunity↑, blood levels of pro-inflammatory factors↓, repair of hippocampal nerve damage, overactivation of microglia and astrocytes↓, GFAP and IBA-1↓	(37)
Flavonoids of *Coreopsis tinctoria* buds	Total flavonoids	Subcutaneous D-galactose	Improvement of body weight in aging mice, brain index in aging mice↑, improvement of spatial learning memory in mice	Oxidative stress damage↓, acetylcholine and antioxidant enzyme activity↑, MDA↓, neuronal cell atrophy↓, amelioration of hippocampal damage in mouse brain tissue	(38)
Anthocyanins in *Ribes meyeri*	Flavanes	Naturally aged 12-month-old mice	Improving spatial cognition in senescent mice	Cellular activity of hippocampal neurons in senescent mice↑	(39)
Nobiletin	Flavone	Subcutaneous injection of D-galactose for 10 weeks induces skeletal muscle atrophy in senescent mice	Amelioration of weight loss in aging mice, narrowing of muscle fibers↓;myofibers are tightly organized	Expression of myogenin, actin↑, activates mTOR/Akt signaling pathway and promotes related protein synthesis	(40)
Orientin and vitexin from *Trollius chinensis* Bunge	Flavone	Intraperitoneal D-galactose	Brain weight in aging mice↑	Oxidative stress damage↓, antioxidant activity↑, harmful free radical ↓, Na^+^-K^+^-ATP and Ca^2+^-Mg^2+^-ATP ↑, lipofuscin↓, significantly improved brain neuronal ultrastructure	(41)
Luteolin	Flavone	In 22–24 months old naturally aged mice, Escherichia coli LPS was injected intraperitoneally 4 weeks after drug administration, and microglia were isolated 4 h later	Proportion of microglia staining for MHC class II, IL-1b, and IL-6 reduced by nearly half	Cerebral microglia activity during aging↓	(42)
Flavonoids from *Rhododendron nivale Hook. f*	Total flavonoids	Subcutaneous D-galactose	Learning memory capacity of aging mice↑	Oxidative stress damage↓, antioxidant enzyme activity in plasma↑, expression of GPx4 in liver and intestine↑	(43)
Flavonoids of white tip silver needle (slightly fermented white tea)	Total flavonoids	Intraperitoneal D-Gal/LPS mixture	Index of each organ in senescent mice↑	Oxidative stress damage↓, antioxidant activity↑, harmful free radical↓, expression of inflammatory factor↓, infiltration of inflammatory factor↓	(44)
Flavonoids of bamboo leaf	Total flavonoids	UV-induced skin aging	Alleviates UV-induced skin aging damage, collagen fiber content↑	Oxidative stress damage↓, antioxidant activity↑, harmful free radicals↓, inflammatory factor secretion↓, MMP-3↓, P62↓, Beclin-1↑, transformation of LC3 I/II in skin tissues↑	(45)
Flavonoids of *Anoectochilus roxburghii*	Total flavonoids	Subcutaneous D-galactose	Learning memory capacity of aging mice↑, improved hepatic histopathological and structural conditions	Oxidative stress damage↓, antioxidant activity↑	(46)
Icariin	Flavonol	Subcutaneous D-galactose	Appearance and mental status of mice↑, learning memory capacity of aging mice↑, P62 mRNA↓, LC3-II, Parkin, Bnip3L↑	Activation of cortical mitochondrial autophagy-related proteins	(47)
Myricetin	Flavonol	Subcutaneous D-galactose	Learning memory capacity of aging mice↑, maintenance of hippocampal neuronal cell morphology	Targeted regulation of the expression of miRs acting on NLRP3, miR-7, miR-138-5p and miR-30e↑, NLRP3, cleavedcaspase-1 (p10)↓, IL-1β, IL-18 and TNF-α↓, activation of the hippocampal NLRP3/Caspase-1 signaling pathway and inflammatory response↓	(48)
Flavonoids of Indocalamus leaves	Total flavonoids	Subcutaneous D-galactose	Antioxidant activity↑, harmful free radical↓	Oxidative stress damage↓	(49)
Hyperoside	Flavonol	Subcutaneous D-galactose	Learning memory capacity in mice↑, exhaustion swimming time in senescent mice↑	Oxidative stress damage↓, antioxidant activity↑, ROS↓, SIRT1, Nrf2 and NQO1↑, regulation of the SIRT1/Nrf2 signaling pathway	(50)
Total flavonoid extract from *Abelmoschus manihot* (L.) Medic Flowers	Total flavonoids	Injected with D-gal	Antioxidant enzyme activity in liver↑, MDA↓, improvement of liver damage	Oxidative stress damage↓, Nrf2, NQO1 and HO-1↑, activation of the Nrf2 signaling pathway	(51)
Chrysin	Flavone	Gavage D-galactose	Learning memory capacity in mice↑, AMPK, LKB1 and PGC1α↑, TNFα, NF-κB, AGEs and GFAP in brain↓, NQO1 and HO-1↑	Regulation of the AMPK/LKB1/PGC1α signaling pathway, oxidative stress damage↓, Neuro factor release↑	(52)
Naringin	Dihydroflavone	Subcutaneous D-galactose	MDA in lung↓, Nrf2 and NQO1 in lung↑, KB1 and AMPK in lung↑, SIRT1/FOXO1↑, Caspase-3 in lung↓, improved alveolar tissue structure	Regulation of the LKB1/AMPK/PGC-1α signaling pathway, FOXO1↑stimulates mitochondrial autophagy↑, oxidative stress injury through activation of the Nrf2/NQO1 pathway↓, amelioration of alveolar space disruption and bronchial wall thickening through the P53/Caspase-3 signaling pathway	(53)
Hydroxysafflor yellow A	Chalcone	Intraperitoneal D-galactose	Body weight of mice↑, spleen thymus index↑, antioxidant activity↑, harmful free radical ↓, hepatocellular inflammatory infiltrate↓, SA-β-Gal in liver↓, P16 and P53 mRNA↓	Oxidative stress damage↓, immunity↑, P16-Rb pathway↓	(54)
Oroxylin A	Flavone	Skin aging caused by UVA and UVB exposure	Amelioration of weight loss in aging mice, skin sensitivity↓, melanin↓, Wrinkles (depth and volume) and skin depressions↓	Oxidative stress damage↓, improve skin collagen alignment	(55)
Taxifolin	Dihydroflavonol	Subcutaneous D-galactose	Improvement of learning memory capacity and cholinergic dysfunction in mice, antioxidant activity↑	Reduction of apoptosis through modulation of Nrf2-mediated oxidative stress and PI3K/AKT pathway	(56)

### 2.3 Anti-aging effects of flavonoids in cellular models

As the fundamental functional unit of human biology, cells represent condensed microcosms of organismal physiology. Cellular senescence triggers senescence-associated secretory phenotype (SASP) expression in critical tissues including heart, liver, and kidneys (57–59). Growing interest in natural anti-aging compounds has expanded research at the cellular level (1). The availability of diverse cellular models and the feasibility of molecular-level mechanistic investigations make this system ideal for anti-aging studies. Flavonoid mechanisms and functional outcomes in cellular models are cataloged in Table 3.

**TABLE 3:** Anti aging effects and mechanisms of natural flavonoids in cell models.

Material	Flavonoid classification	Cell model	Modeling method	Effect	Mechanism	References
Procyanidin C1	Dihydroflavonol	Primary normal human prostate stromal cell linePSC27, human foetal lung fibroblasts (WI38), primary human umbilical vein endothelial cells (HUVECs) and human mesenchymal stem cells (MSCs)	Sublethal doses of bleomycin treatment	Removing senescent cells	Senescent cell apoptosis↑, no effect on proliferating cells, p16 positive cells↓, mitochondrial dysfunction in aging cells↑	(60)
Galangin	Flavonol	Primary neonatal human foreskin dermal fibroblasts	40 mJ/cm^2^ UVB radiation induced 24 h	Ameliorated UVB-induced decrease in cell viability, number of SA-β-gal positive cells↓, ROS↓	SIRT1↑, UVB-induced P53 acetylation and nuclear translocation↓, apoptosis↓	(61)
Myricetin	Flavonol	MLE12 cells	100 μM Silicon dioxide incubation for 2 h	Silicon dioxide-induced cellular senescence↓, β-gal↓	P53, P21↓, IL-6, IL-1β and MMP-9 mRNA↓, mitochondrial autophagy↑, PPARγ and PGC-1α↑	(62)
Oroxylin A	Flavone	L02 cell	i.g. with 56% ethanol, 5 mL/kg, once every 2 days.	Restorative effect on cell viability, AST, ALT and LDH levels in cell supernatants after ethanol treatment↓;number of SA-β-gal-positive cells↓, elimination of ethanol-induced G0/G1 cell-cycle blockade	P16, P21 and γ-H2AX↓, YAP↑, telomerase activity↑, telomere length↑	(63)
Luteolin	Flavone	SW1353 Cells	50 μM Tert-butyl hydroperoxide induced 24 h	Number of SA-β-gal positive cells↓, G0/G1 cell cycle block↓	RNA and protein levels of P53/P21 and MMP13↓, COL2A1 and CDK2/CDK4 RNA and protein levels↑, alleviating cell cycle arrest and proliferation stagnation	(64)
Genistein	Flavone	OVX-BMMSC	From postmenopausal women and ovariectomized (OVX) rodents	ROS↓, mitochondrial dysfunction↓	SIRT3 and PGC1α↑, maintain mitochondrial homeostasis and restore mitochondrial function	(65)
Flavonoids of bamboo leaf	Total flavonoids	HaCaT cell	Induced by oxidant 1 mM AAPH	Oxidative stress↓, anti-inflammatory, antioxidant↑	P21, P16 and K9M-H3↓, regulation of P38 MAPK signaling pathway	(45)
Flavonoids of *Anoectochilus roxburghii*	Total flavonoids	L02 cell	400 μmol/L H_2_O_2_ induced	Antioxidant activity↑, harmful free radical↓, mitigation of cellular damage	Oxidative stress damage↓	(46)
Flavonoids of cocoa	Total flavonoids	House Ear Institute-Organ of Corti 1 (HEI-OC1), stria vascularis (SV-k1), and organ of Corti (OC-k3) cells	100 μM H_2_O_2_ induced for 1 h	H_2_O_2_-induced impairment of auditory cell viability↓, ROS/RNS in senescent auditory cells↓, prevention of DNA damage in auditory senescent cells	Oxidative stress damage↓, caspase-3↓, ATP↑, mitochondrial apoptosis↓	(66)
Icariin	Flavonol	HUVECs	Homocysteine induced for 3 days	Proportion of SA-β- gal positive cells↓,ROS↓	Phosphorylation of AKT and ERK↑, NO↑, activate the PI3K/AKT signaling	(67)
Apigenin	Flavone	Human embryonic lung fibroblasts(WI-38 cells)	200 μM H_2_ O_2_ or 100 ng/ml DOXO	Proportion of SA-β-gal positive cells↓, NAD^+^ and NAD^+^/NADH↑	ac-P53, P21^Waf1Cip1^ and P16^Ink4a^↓, p-Rb and cyclin D1 protein level↑, SIRT1↑, CD38 siRNA↓, modulation of the P53- P21^Cip1WAF1^ and P^16Ink4a^-Rb pathways	(68)
Total flavonoids in stems and leaves of *Glycyrrhiza uralensis* Fisch.	Total flavonoids	HT-22 cell	2 μg/mL LPS induced for 24 h	LPS-induced apoptosis↓, antioxidant activity↑,MDA↓, ROS↓	Oxidative stress damage↓, MMP↓, Ca^2+^↓, improving mitochondrial Ca^2+^ dysregulation	(21)
Baicalein	Flavone	NPCs	100 μmol/L tert-butyl hydroperoxide induced for 4 h	β-gal↓, TNF-α↓, P53, P16, IL-1β, TNF-α, MMP-3, MMP-9↓	Inflammatory factor release↓, expression of markers of aging↓	(69)
Hesperetin	Dihydroflavone	HEK001 Human keratinocytes	Obtained from elderly subjects	Mitochondrial oxygen consumption in cells↑, mitochondrial function↑, UV-induced cellular damage↓, MMP-1↓	Oxidative stress damage↓, expression of iron-sulfur-containing structural domain protein 2 (CISD2)↑	(70)
Oroxylin A	Flavone	HaCaT cell	60 mJ/cm^2^ UVB irradiation for 24 h	Antioxidant activity↑, ROS↓, apoptosis↓	Modulation of the SIRT1/Nrf2 signaling pathway reduces apoptosis	(55)

Our summary shows that flavonoids exhibit significant anti-aging activity in C. elegans, animal and cellular aging models. The advantages and disadvantages of each model are summarized in Table 4.

**TABLE 4:** A comparison of the advantages and disadvantages of C. elegans, animal and cell models.

Model	Advantages	Shortcomings
C. elegans	It has a short lifespan and is suitable for high-throughput drug screening. It is also genetically stable, simple to operate and low cost.	It is impossible to fully simulate the chronic diseases caused by aging in humans. This makes it difficult to translate research results into clinical applications, and to gain a comprehensive understanding of the mechanisms of higher animal tissue systems.
Animals (rats/mice)	It can simulate the complex interactions between multiple tissues and organs that occur during human aging as accurately as possible. It can also simulate various age-related diseases, such as Alzheimer’s and cardiovascular disease. These complex physiological processes are invaluable, and the research findings can provide a robust foundation for clinical practice.	The research subjects varied greatly, resulting in unstable results. The trial was not repeatable, the operating environment requirements were high, the costs were high, and the cycle was long.
Cells	It is also suitable for high-throughput drug screening and can monitor organelle-level senescence. It has a short experimental cycle and simple modelling methods, which make it conducive to studying drug molecular mechanisms.	Comprehensive expression is difficult to achieve, such as the lack of differences in expression between various tissues and organs. The transformation process into higher animals has a high failure rate. It is impossible to express the long-term and spatial effects of higher animal models.

### 2.4 Other models and roles

Alternative model systems further demonstrate flavonoids’ anti-aging potential. Pu et al. (71) utilized network pharmacology to predict anti-aging mechanisms in Lantana camara, identifying quercetin, apigenin, baicalein, kaempferol, naringenin, and lignans as key components that modulate aging- and tumor-related pathways. Li et al. (72) reported potent *in vitro* antioxidant activity in Ginkgo biloba seed kernel extracts, suggesting anti-aging effects requiring further validation. In yeast models, sacred lotus stamen flavonoids extended the chronological lifespan of Saccharomyces cerevisiae DBY746 by enhancing mitochondrial function, antioxidant enzymes, and NADH/ATP production (73). Concurrently, Guo et al. (74) found neohesperidin prolonged stationary-phase survival in S. cerevisiae BY4742 following nutrient depletion, though its ROS-scavenging capacity was limited and mechanisms remain uncharacterized. Complementing these findings, Lycium ruthenicum (black wolfberry) flavonoid extract significantly extended Drosophila melanogaster lifespan by alleviating H_2_O_2_-induced oxidative damage (35).

## 3 Study on the mechanism of anti-aging effects of natural flavonoids

### 3.1 Free radical scavenging mechanisms

**FIGURE 4:**
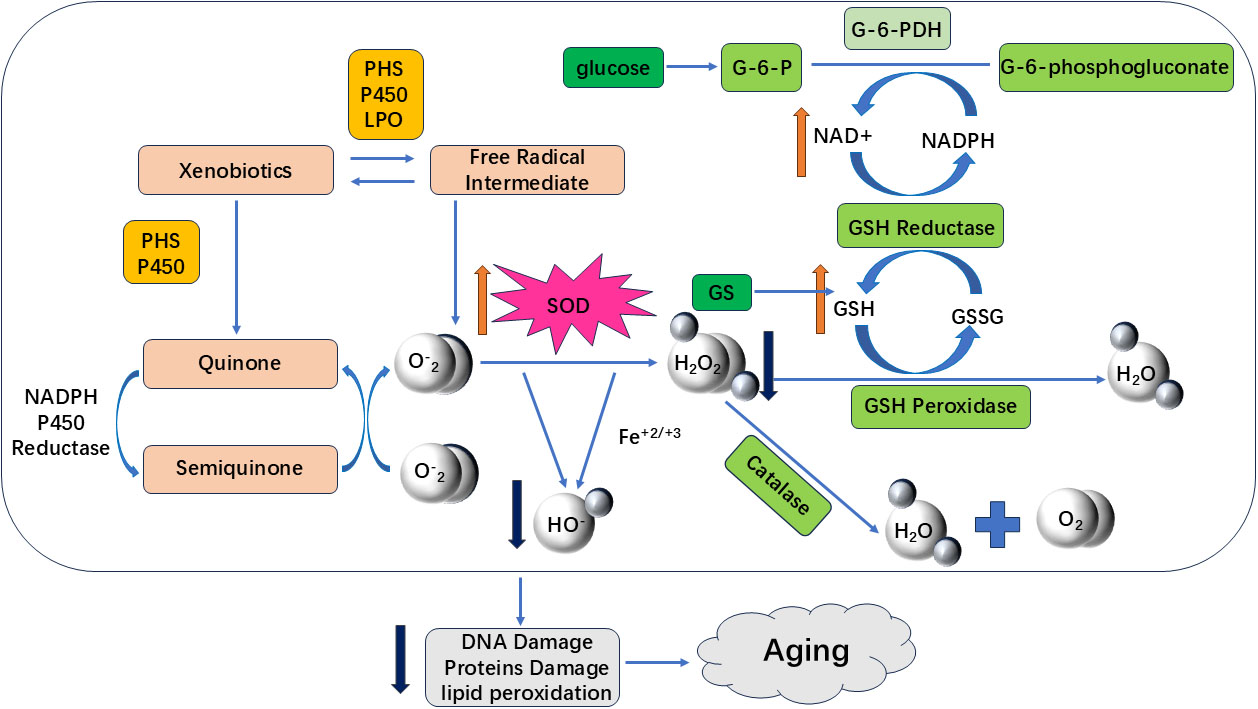
Aging induced by oxidative stress. The red upward arrow in the figure indicates an increase in antioxidant enzyme activity, a decrease in ROS and harmful free radical accumulation, and a delay in aging; The dark blue downward arrow indicates a reduction in harmful free radical content or oxidative stress damage, delaying aging.

Human physiological processes inherently generate oxidative reactions. An imbalance between oxidation and antioxidant defenses induces oxidative stress, producing harmful free radicals that damage cellular components. Chronic oxidative stress causes cumulative DNA, protein, and tissue damage, accelerating aging and disease pathogenesis (75). Most flavonoids counteract aging through antioxidant mechanisms - reducing oxidative stress and enhancing antioxidant capacity across model systems including C. elegans, cellular models, and D-galactose-induced animal models. This aligns with Harman’s Free Radical Theory of Aging (1956), which posits that age-related accumulation of free radicals drives DNA damage, metabolic dysregulation, and degenerative conditions including neurodegeneration and cancer (76–78). As illustrated in Figure 4, flavonoids reduce oxidative stress-induced damage by enhancing the activity of antioxidant enzymes, such as SOD and GSH. They do this by eliminating excess ROS and H_2_O_2_, which are harmful free radicals, and by regulating redox balance to exert anti-aging effects.

### 3.2 Nutrient-sensing pathway regulation

**FIGURE 5:**
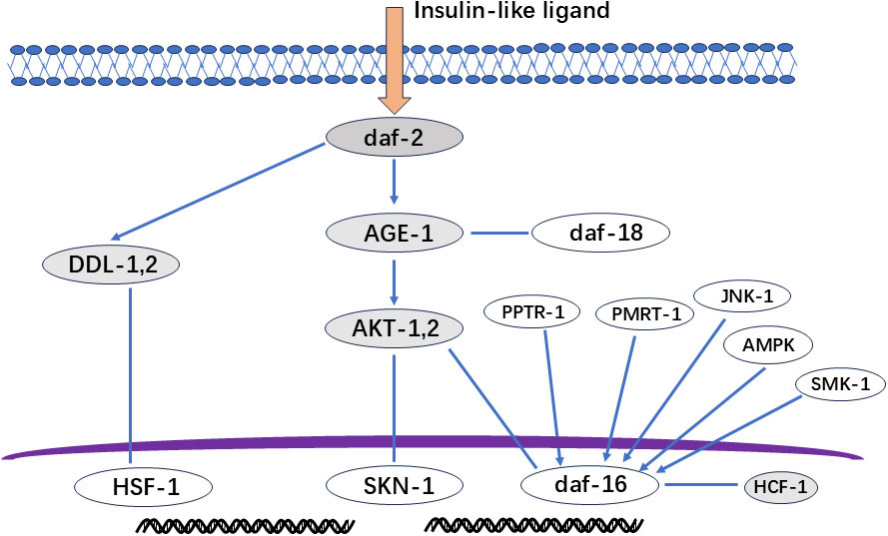
IIS signal regulation diagram. Gray section: The deletion mutation of daf-2 (insulin-like growth factor receptor) can cause long-term nematode aging and death; Age-1 (3-Phosphatidylinositol Kinase): Mutation prolongs lifespan; AKT-1 and AKT-2 (protein kinase): daf-16 not phosphorylated by AKT will be transported to the nucleus to regulate the expression of downstream genes and play a role in prolonging life; DDL-1, 2: The heat shock factor HSF-1 forms a trimeric DHIC with two other proteins, DDL-1 and DDL-2, but HSF-1 needs to dissociate from DDL1,2 to form a monomer for activation activity; HCF-1: A protein highly homologous to host cell factor 1 in C. elegans, but HCF-1 interacts with daf-16 to prevent daf-16 from binding to the promoter of downstream genes regulating aging. Therefore, knocking down HCF-1 can stimulate daf-16 to exert anti-aging effects.

Beyond oxidative stress mitigation, flavonoids extend lifespan in C. elegans primarily through insulin/insulin-like signaling (IIS) pathway modulation (Figure 5) (79). This regulatory pathway is similar to those found in higher organisms. In Caenorhabditis elegans, under nutrient-rich conditions, insulin-like growth factor ligands bind to receptors. This triggers a series of protein kinase cascades, resulting in the phosphorylation and modification of DAF-16 by AKT-1 and AKT-2. DAF-16 that has been phosphorylated by AKT cannot enter the nucleus and is inactivated as a transcription factor. However, in DAF-2 or AGE-1 mutant C. elegans, this cascade is not activated and unphosphorylated DAF-16 is transported into the nucleus. There, it regulates the expression of downstream genes, thereby extending lifespan. The key regulatory factors DAF-2 and DAF-16 control the expression of downstream genes during the dauer larval stage. Flavonoids can enhance their anti-aging functions. Key regulators DAF-2 and DAF-16 control downstream effectors during the dauer larval stage (80, 81), flavonoids enhance their anti-aging functions by increasing the expression of daf-2 and daf-16 (82). While these genes respond to multiple factors (Figure 5), further research is needed to elucidate: (1) their downstream regulatory mechanisms, and (2) their role in nutrient-sensing mediated lipid/glucose metabolism.

### 3.3 Sirtuin pathway activation

**FIGURE 6:**
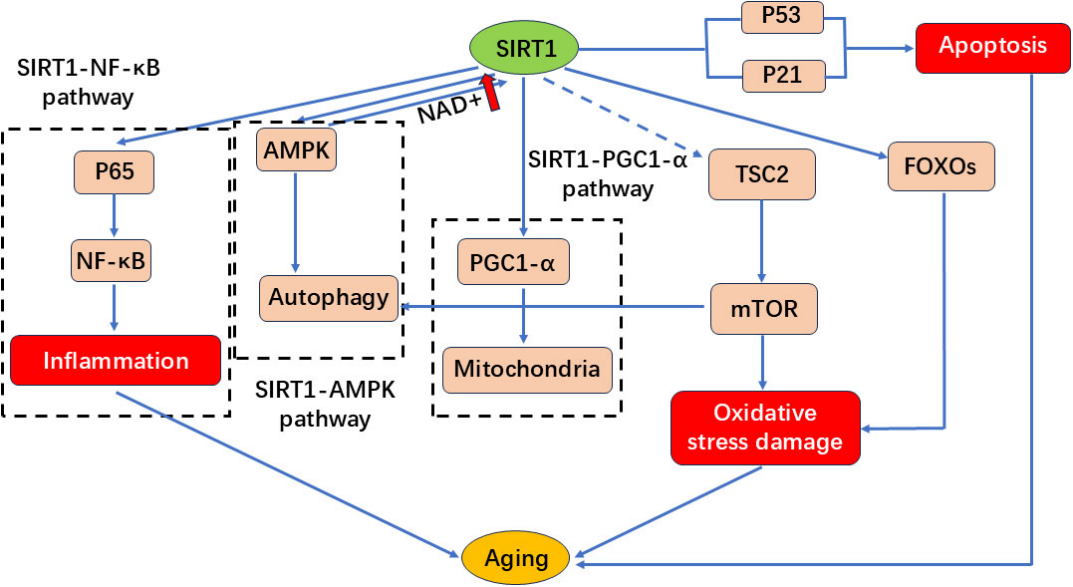
SIRT1 regulates the signaling pathway diagram.

Sirtuins (NAD^+^-dependent deacylases) sense metabolic cues, modulate mitochondrial function, exert neuroprotection, and mitigate age-related pathologies (83). SIRT1 activation has emerged as a promising therapeutic strategy for delaying aging and improving metabolic health (84–86). As depicted in Figure 6, flavonoids combat cellular senescence by enhancing SIRT1 expression and activity (87). In the SIRT1-NF-κB signaling pathway, P65 acetylation enhances NF-κB activity, triggering inflammatory responses and leading to aging. However, SIRT1 can inhibit NF-κB activity by mediating P65 deacetylation, thereby reducing inflammation and delaying the aging process. In the SIRT1-AMPK signaling pathway, AMP-activated protein kinase (AMPK) plays a key role in cellular energy metabolism and survival. AMPK activation can enhance autophagy and prolong lifespan. SIRT1 interacts with AMPK and can enhance its own activity by increasing NAD^+^ levels. In the SIRT1-PGC1α signaling pathway, peroxisome proliferator-activated receptor coactivator-1α (PGC-1α) is the primary transcriptional coactivator responsible for regulating mitochondrial function and maintaining mitochondrial homeostasis. SIRT1 regulates mitochondrial function and metabolic balance by increasing mitochondrial biosynthesis and oxygen consumption through the deacetylation of PGC1α, thereby delaying aging. It has been shown that inhibiting mTOR activity can prolong the lifespan of various organisms. SIRT1 delays aging by blocking the mTOR pathway and activating autophagy. SIRT1 can also interact with tuberous sclerosis protein 2 (TSC2), an upstream inhibitor of TORC1 that negatively regulates mTOR signaling in a TSC2-dependent manner. P53 activation is involved in cell apoptosis, cell cycle arrest and aging. SIRT1 leads to the deacetylation of P53, thereby inhibiting DNA damage and stress-induced cell aging. P21 is a P53 target, and reducing the expression of both P53 and P21 can delay aging. FOXOs are a family of proteins that act as sensors in the insulin signaling pathway and are a type of transporter protein. They are involved in oxidative stress, DNA damage repair, autophagy and cell cycle arrest. FOXOs bind to SIRT1 to regulate cellular aging. In mammalian cells, SIRT1 reduces oxidative stress damage by regulating FOXOs.

### 3.4 Other mechanism of action

Flavonoids combat aging through multifaceted mechanisms beyond oxidative stress mitigation and nutrient-sensing pathway modulation. Aging hallmarks—including genomic instability, telomere attrition, epigenetic alterations, proteostasis collapse, impaired autophagy, dysregulated nutrient sensing, mitochondrial dysfunction, cellular senescence, stem cell exhaustion, and chronic inflammation (88)-are counteracted by flavonoids via apoptosis regulation, suppression of inflammatory factor release, enhanced autophagic flux, and telomerase activation. Critically, flavonoids exhibit pleiotropic regulation, with single compounds concurrently engaging mTOR, IIS, and AMPK pathways. For instance, mTOR signaling modulates MAPK expression and senescence-associated secretory phenotypes (SASP), integrating these pathways into a coordinated anti-aging network (89–91). While flavonoids demonstrably ameliorate aging phenotypes across cellular, tissue, and organismal levels, their comprehensive mechanistic landscape remains an active research frontier.

## 4 Discussion

The continuous growth of the global elderly population imposes substantial pressure and poses significant challenges for society, the economy, and service industries. Developing natural and effective anti-aging drugs and health products represents a promising approach to mitigate this issue. This paper reviews the anti-aging effects of flavonoid compounds—a subject of considerable research interest. By synthesizing available data, we summarize the roles of flavonoids in aging across model organisms (e.g., *Caenorhabditis elegans*), animal models, and cellular systems. From both *in vivo* and *in vitro* perspectives, this work elucidates the value and potential of flavonoid compounds in anti-aging research.

In summary, future research on flavonoid anti-aging effects requires optimization in several areas. First, isolating and identifying individual compounds remains time-consuming; most current studies focus on total flavonoids, with limited reports on single constituents. Subsequent studies should employ advanced chromatographic techniques for efficient compound separation and analysis to better understand their chemical profiles and anti-aging properties. Second, current models primarily utilize C. elegans, mice, and cells, while organisms like Drosophila and yeast are underutilized—likely due to higher costs and specialized expertise requirements for the latter. Future efforts should increase investment, implementing a three-tiered “model organisms-animals-cells” strategy combined with *in vivo*/*in vitro* approaches for comprehensive validation. Third, most animal aging models rely on D-galactose injection, whereas naturally aged or genetic defect models are scarce due to long experimental cycles, multifactorial instability, and high costs. More cost-effective, operationally simple modeling methods are needed to enhance scientific rigor. Regarding mechanisms, current research emphasizes the free radical theory targeting oxidative stress reduction. However, as oxidative stress is not the sole driver of aging, future studies should adopt integrated strategies—combining genomics, proteomics, and multi-level (cellular/tissue) analyses—to identify novel targets and signaling pathways, thereby establishing a holistic understanding of anti-aging mechanisms.
